# Recombinant anti-vascular endothelial growth factor fusion protein efficiently suppresses choridal neovasularization in monkeys

**Published:** 2008-01-10

**Authors:** Ming Zhang, Junjun Zhang, Mi Yan, Hong Li, Chun Yang, Dechao Yu

**Affiliations:** 1 Department of Ophthalmology, West China Hospital, Sichuan University, China; 2 Chengdu Kanghong Biotechnology Co. Ltd, Sichuan Province, China

## Abstract

**Purpose:**

KH902 is a fusion protein which combines ligand binding elements taken from the extracellular domains of vascular endothelial growth factor (VEGF) receptors 1 and 2 and the Fc portion of IgG1. This study is designed to examine the inhibitory effect of KH902 in the choroidal neovascularization (CNV) monkey model.

**Methods:**

The binding affinity with VEGF was measured by using the human VEGF ELISA kit, and the biological activity effect of KH902 was assayed by an in vitro inhibition experiment on human umbilical vein endothelial cell proliferation that was induced by VEGF. The experimental CNV was induced by causing perimacular laser injury in the eyes of rhesus monkeys and confirmed by fluorescence fundus angiography (FFA), optical coherence tomography (OCT), and multifocal electroretinograms (mf-ERG) 20 days after the infliction of the laser injury. KH902 was delivered to the animals through intravitreal injection at various doses. Monkeys were observed four weeks after injection by ophthalmic examination, FFA, OCT, mf-ERG, histopathology, and immunohistochemistry analysis.

**Results:**

KH902 binds VEGF at a high affinity with a mean of IC_50_ of 10 pM. KH902 at 41 nM can completely block VEGF-induced cell proliferation and KH902 at 10.7 nM can block 82.6% of cell growth. In the eyes of the treatment group, which received 300 μg and 500 μg KH902, choroidal neovascularization leakage was obviously less than before injection, and no leakage was observed at the end of the observation after injection. No high reflect light echogenic mass was detected by OCT. However, in the 0.1 mg KH902-treated and control eyes, the leakage and high reflect light echogenic mass still existed. The reduction of experimental CNV was greater in eyes treated with 300 μg and 500 μg KH902 than in eyes treated with 0.1 mg KH902 and the control eyes. There were fiber-vasculosa membrane proliferation in the 100 μg KH902-treated eyes and control eyes but not in the 300 μg and 500 μg KH902-treated eyes under histopathologic observation. The results of mf-ERG demonstrated that there was greater improvement in the 300 μg and 500 μg KH902-treated eyes than in the 100 μg KH902-treated eyes and control eyes.

**Conclusions:**

KH902 presents high affinity with VEGF and inhibitory activity on the proliferation of human umbilical vein endothelial cells (HUVECs) induced by VEGF. A single 300 μg or 500 μg KH902 intravitreal injection effectively inhibited leakage and growth of the CNV in rhesus monkeys without evidence of toxicity. This study suggests that KH902 has promise as a local antiangiogenic treatment of CNV.

## Introduction

Age-related macular degeneration (AMD) is the leading cause of irreversible blindness among people who are 50 years of age or older in the developed world [1,2]. The choroidal neovascularization (CNV) accounts for the severely progressive decrease of central visual acuity among 90% of neovascular/exudative (wet) AMD [3]. Although the etiological factors and pathology process of CNV still remain elusive, vascular endothelial growth factor (VEGF)-A is a major regulator of angiogenesis and vascular permeability implicated in the development of the CNV involving pathological angiogenesis and increased vascular permeability [4,5]. Multiple biologically active forms of VEGF-A are generated by alternative mRNA splicing and proteolytic cleavage, and two isoforms have been detected in choroidal neovascular lesions [6].

Although conventional laser photocoagulation [7] and photodynamic therapy (PDT) [8] were applied as prevalent therapeutic modalities for CNV, a small percentage of patients with CNV fit the conventional laser and usually the visual outcome was poor after the treatment. Furthermore, over half of the treated patients with CNV have a recurrence after laser [9]. Verteporfin PDT (Visudyne; Novartis, East Hanover, NJ) has been shown to stabilize or slow down vision loss in patients with neovascular AMD but requires repeated treatments which may be associated with cumulative damage to normal retinal structures [10]. Neither of these options are effective for all patients with neovascular AMD, and improved or even stabilized visual acuity (VA) is not commonly achieved even with treatment. Recently, the therapies that aimed at VEGF-A as the target for the management of CNV associated with AMD have demonstrated encouraging signs of biologic activity with confirmed efficacy and safety such as the intravitreal injection of Pegaptanib [11] and Ranibizumab [12]. But the half-life of them is less than four days after nonclinical vitreous administration, repeated intravitreal injections were needed, which increased the risks of endophthalmitis and retina detachment [12].

KH902 is a humanized fusion protein, which is aimed to bind to all forms of VEGF-A. It can efficiently bind VEGF and can theoretically inhibit proliferation of endothelial cells. The present study was designed to demonstrate the binding affinity with VEGF and efficacy of KH902 in the suppression of the experimental CNV in the rhesus monkey.

## Methods

KH902 is an engineered protein which contains the extracellular domain 2 of vascular endothelial growth factor receptor (VEGFR) 1 (Flt-1) and extracellular domain 3,4 of VEGFR2 (KDR-3,4) fused to the Fc portion of human immunoglobulin G1. The molecular weight is about 143 kDa.

### Vascular endothelial growth factor receptor binding assay of KH902 and human umbilical vein endothelial cell proliferation assay

Binding affinity with VEGF was measured by using the human VEGF ELISA kit (DY293B; R&D, Minneapolis, MN) for detecting free human VEGF in mixtures of KH902 (concentration ranging from 0.1 pM to 0.5 nM) with human VEGF. And the affinity was evaluated by IC_50_. IC_50_ was calculated by the corresponding concentration of KH902 with half of free VEGF. To determine the binding affinity of KH902 with VEGF, binding assays were performed in which different concentrations of KH902 were incubated with VEGF165 and the method was in accordance with the ELISA kit.

To evaluate the bioactivity of KH902 in vitro, the proliferation of human umbilical vein endothelial cells (HUVECs) was taken as a good index. HUVECs were cultured in conditioned medium containing 0.2 nM VEGF and serial concentrations of KH902 (41 nM, 10.7 nM, and 0 nM). The control group was medium without VEGF and KH902. Four days later, 15 μl CCK-8 (Dojindo Molecular Technologies,Kumamoto, Japan) was added into each well and the absorbance was measured at 570/630 nm after 2 h.

### Animals and laser-induced choroidal neovascularization

Rhesus monkeys were used in accordance with the Association for Research in Vision and Ophthalmology (ARVO, Rockville, MD) resolution on use of animals in research and in compliance with guidelines developed by the Animal Care Committee of the Sichuan University (Chengdu, China). Monkeys weighed between 2 and 5 kg, and ages ranged from three to six years. For the experimental procedures, animals were anesthetized with 2.5% soluble pentobarbitone (1 ml/kg). Supplemental anesthesia was given with 2.5% soluble pentobarbitone (0.8 ml/kg). Topical ocular anesthesia was obtained with proparacaine.

CNV was induced by a laser (Vissulus 532s Laser Photocoagulator, Carl Zeiss Meditec AG, Jena, Germany). Laser photocoagulations were conducted to the perimacular region of monkey eyes. Lesions were placed in the macula with eight spots. Laser lesions were placed in a circular fashion around the macula about one disk diameter from the foveal center. Care was taken to avoid lasering the fovea. The approximate laser parameters were as following: spot size, 50 μm; laser power, 300–500 mW; and exposure time, 0.05 s. Color photographs were taken on the day of the laser procedure. The ultimate laser power used was determined by the appearance of a small blister and the sound of a pop indicating a puncture of Bruch's membrane. If no blister or pop was noted, a second laser spot was placed over the initial spot.

### Intravitreal injection of KH902 and control

Twenty days after the laser burn, monkeys were divided into two groups, the KH902 treatment group and the control group, according to the results of fluorescence fundus angiography (FFA) and optical coherence tomography (OCT). The eyes of the treatment group received three doses of KH902 (500 μg, 300 μg, and 100 μg) and the control group received an intravitreal vehicle injection. Before the intravitreal injection, eyes of the monkeys were administrated tobramycin drops in the fornices over eight times. After the animal was anesthetized, the eye was anesthetized with a drop of proparacaine in the conjunctival sac and the pupil was dilated by 0.5% tropicamide eye drops (Mydrin P; Santen Pharmaceutical, Osaka, Japan). A 5% povidone iodine solution was placed in the conjunctival sac. A self-retaining eyelid speculum was placed in the eye. We used calipers to measure and mark a location 2–3 mm behind the limbus. We used forceps to stabilize the eye and conducted the intravitreal injection with a 30-gauge needle. The needle was visualized in the pupil and the drug or placebo was injected into the midvitreous. We withdrew the needle and instilled erythromid ointment in the fornices.

### General ophthalmologic examination

Ophthalmologic examinations were performed before laser and followed the scheduled examination time during the entire study period. The fundus, anterior segment, and intraocular pressure (IOP) were examined by indirect ophthalmoscopy, slit-lamp microscopy, and Tono-Pen tonometer in both eyes. The animals were lightly sedated with ketamine hydrochloride before this procedure, and a few drops of 0.5% tropicamide and 0.4% oxybuprocaine hydrochloride (Benoxil.RTM. 0.4% solution; Santen Pharmaceutical) were instilled into each eye to facilitate the examination.

### Multifocal electroretinogram analysis

Multifocal electroretinograms (mf-ERGs) were obtained before laser treatment and 20 days after the infliction of the laser injury. After the intravitreal injection of KH902, the same procedure was applied on days 14 and 28. Mf-ERGs were recorded by a multifocal electroretinograph diagnostic system (Roland Consult Elektrophysiologische Diagnostik Systeme, Retiscan multifocal ERG Version 3.2, Brandenburg, Germany) with what is often referred to as the “standard” stimulus paradigm. The “standard” visual stimuli are comprised of an array of 61 hexagonal elements displayed on a 21-inch monochrome cathode ray tube at a 75 Hz frame rate. Approximately 30 min before recording, pupils of each animal were dilated to about 6 mm and sedated with intravenous 2.5% soluble pentobarbitone (1 ml/kg). A mydriatic was instilled in each eye approximately 10–15 min before the mf-ERG procedure and the animal was placed in a prone position. An intravenous 1 mg/kg 2.5% soluble pentobarbitone was given to maintain sedation. Mf-ERG probes were placed subcutaneously beneath each eye and another on top of the head posterior to the brow. Lubricant (carboxymethylcellulose) was applied to each lens, and contact lenses were placed on each eye. The mf-ERG tests were repeated at least five times. After completion of the test, tobramycin drops was placed in each eye.

### Optical coherence tomography examination

Under sedation, the animals were fixed to keep eye open and maintain the position of the head. An OCT scan was applied with Stratus OCTTM model 3000 (Carl Zeiss Medited Inc., Dublin, CA). When fixed on the macular fovea by observation on the monitor screen, the fast macular scan procedure was applied to check each eye of the monkeys before the laser injury and 20 days after the injury. On days 14 and 28 after intravitreal injection, the same OCT examination was applied again. The analysis was kept the same by choosing the equal scan angle.

### Color photography and fluorescein fundus angiography

Color photography and fluorescein fundus angiography (FFA) were performed before laser treatment and 20 days after laser treatment. After intravitreal injection of the KH902, color photography and FFA were performed on days 14 and 28. The animals were sedated with intravenous 2.5% soluble pentobarbitone (1 ml/kg). Eyelids were fixed to keep the eye open. Each animal was placed on an ophthalmology restraint stand to maintain the position of the head during photography. Photographs were taken by a fundus camera (FF450 plus IRu Retina Camera, Software Visupac version 3.5, Carl Zeiss Meditec AG, Jena, Germany). Before the administration of fluorescein dye, color photographs were taken first. Fluorescein dye (20% fluorescein sodium; 0.05 ml/kg) was then injected via a vein of the lower extremity. Photographs were taken at several time points after the dye injection including the arterial phase, early arteriovenous phase, and several late arteriovenous phases to monitor leakage of fluorescein associated with CNV lesions. Color fundus photography and fluorescein angiography were used to detect and measure the extent and evidence of leakage of CNV. These were performed in a masked fashion by two of the authors (Junjun Zhang and Mi Yan, Department of Ophthalmology, West China Hospital, Sichuan University, Chengdu, China). Angiographically, the burn is hypofluorescent early. If CNV is present, hyperfluorescence develops around the burn, which progresses to late diffuse leakage with dye pooling in the serous detachment surrounding the burn area. The basis for this determination was graded by the degree of the leakage on a standardized scale of 1–4. Grading scores were defined as the followings: 1, no hyperfluorescence; 2, hyperfluorescence without leakage; 3, early hyperfluorescence and late mild leakage; and 4, early hyperfluorescence and late severe dye leakage beyond the borders of the burn area. A grade of 4 was assigned to the clinically significant fluorescence leakage of the classic experimental CNV. The area of the late grade 4 CNV lesion leakage was measured with software (Software Visupac version 3.5; Zeiss Corporation) in a masked fashion by two of the authors (Junjun Zhang and Mi Yan, Department of Ophthalmology, West China Hospital, Sichuan University). Data were analyzed by SPSS 13.0.

### Histopathologic and immunohistochemistry analysis

Animals were killed with intravenous veterinary pentobarbitol-based euthanasia solution (Vortech Pharmaceuticals, Dearborn, MI). The globes were carefully removed from each animal and dissected clean of orbital tissue. The globes were rinsed in saline and placed in modified fixative consisting of 1.5% glutaraldehyde and 2.5% formaldehyde in 0.1 M phosphate buffer, 7.4 pH. Four hours later, we opened a 5 mm diameter “window” near the limbus wall of globes. Another “window” opposite to the first one were made after one night. The globes were then placed in a modified fixative for 24–48 h. The anterior segment was dissected and discarded. The posterior pole was changed to a buffer (0.1 M phosphate) until processed for serial section. A 3 μm section that contained lesions of interest was prepared to hematoxylin and eosin-staining and CD31 and CD105 immunohistochemistry staining for analysis.

The paraffin sections were soaked in dimethyl benzene and gradient alcohol for deparaffinage then digested with 0.1% trypsin to repair the antigen (for 0.5 h at 37 °C). The sections were incubated with CD31 monoclonal antibody (Rabbit antibody, dilution 1:300) and CD105 monoclonal antibody (Mouse antibody, dilution 1:100,) for 2 h at 37 °C. Normal rabbit serum (dilution 1:100 in PBS) was used as a negative control. Washing was performed with PBS, and sections were incubated with the secondary anti-mouse antibody conjugated with FITC (dilution 1:50; Cappel, Durham, NC) and the anti-rabbit antibody conjugated with tetramethyl rhodamine isothiocyanate (TRITC; dilution 1:200; Cappel). After incubation for 2 h at 37 °C, washing was performed with PBS. Sections were mounted with DAPI mounting medium and observed with a fluorescence microscope (Nikon TE2000-U; Nikon Co, Ltd., Tokyo, Japan).

### Statistical analysis

Data are presented as mean±SD and analyzed by SPSS 13.0 software. The difference among the means of the groups is determined with ANOVA. When a significant difference is determined, the Dunnett post hoc analysis is used. p<0.05 was considered significant.

## Results

### KH902 high binding affinity to vascular endothelial growth factor

**Figure 1:**
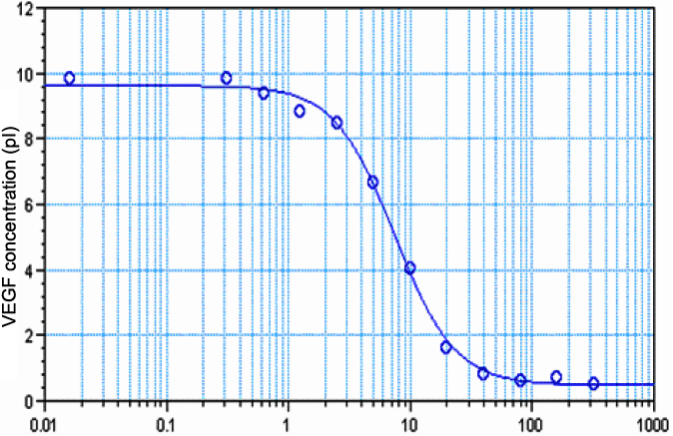
Binding affinity of KH902 for vascular endothelial growth factor. Affinities of indicated KH902 for VEGF was determined by using a binding assay that measures free VEGF (ordinate) after incubation of 10 pM of human VEGF165 with varying concentrations of KH902 (abscissa).

The amount of unbound VEGF165 was measured, revealing that the IC_50_ of KH902 was between 7 and 15 pM. We repeated the binding assay several times with the results shown in Figure 1. As shown in the results, KH902 has a high affinity with VEGF, and the mean IC_50_ was 10 pM.

### KH902 blocks vascular endothelial growth factor-induced human umbilical vein endothelial cell proliferation

**Figure 2:**
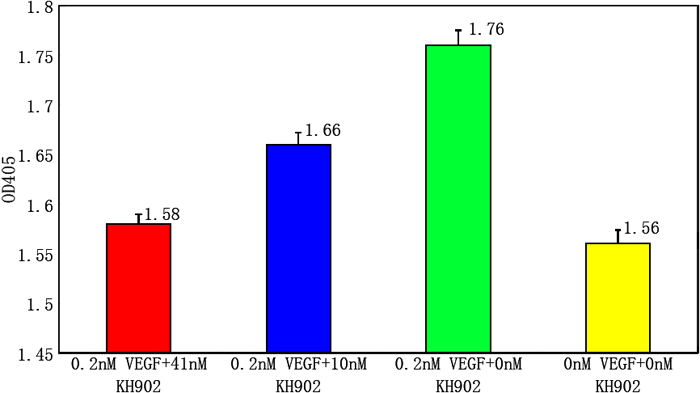
The effect of KH902 on vascular endothelial growth factor-induced human umbilical vein endothelial cell proliferation. KH902 effectively inhibits VEGF-induced HUVEC proliferation. The medium containing 0.2 nM VEGF increased cell growth of HUVEC. However, KH902 presents the inhibition on cell growth at a concentration of 10 nM. It can also almost completely block the VEGF-induced effect at 41 nM.

The growth of endothelial cells are induced by a lot of factors including VEGF. To determine whether KH902 binding of VEGF could potently and effectively block the ability of VEGF to enhance endothelial cells proliferation, VEGF and KH902 were added to HUVECs and the effect on proliferation was examined. The results as shown in Figure 2 demonstrate that KH902 at 41 nM can completely block VEGF-induced cell proliferation, and KH902 at 10.7 nM can block 82.6% of cell growth (Figure 2).

### KH902 inhibits the growth and leakage of experimental monkey choroidal neovascularization

**Figure 3:**
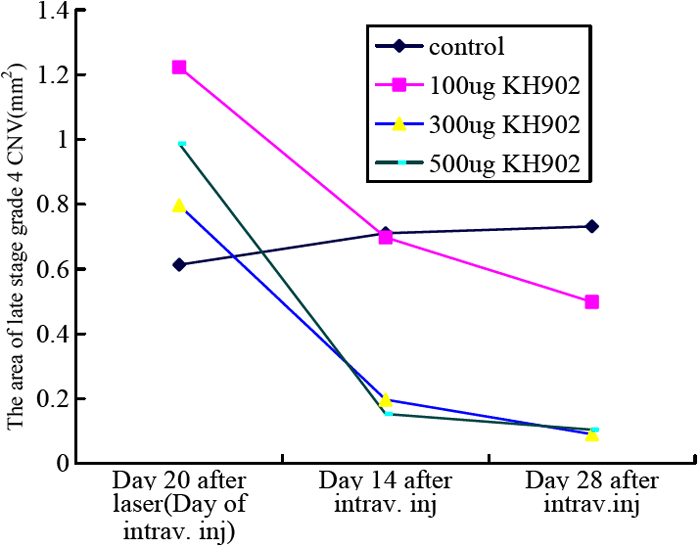
Mean area changes of grade 4 spots on day 20 after laser, day 14 and day 28 after intravitreal injection in control, 100 μg, 300 μg, and 500 μg KH902-treated groups. CNV spots were graded on a scale of 1–4 20 days after laser and on days 14 and 28 after intravitreal injection. The bigger area of grade 4 spots represented greater CNV leakage. The area of neovascularization was significantly less on days 14 and 28 than on day 20 after laser in the 300 μg and 500 μg KH902-treated eyes. Furthermore, the area was significant lower in the 300 μg and 500 μg KH902-treated eyes than in the control and 100 μg KH902-treated eyes (ANOVA, p<0.001). Grading scores were defined as follows: 1, no hyperfluorescence; 2, hyperfluorescence without leakage; 3, early hyperfluorescence and late mild leakage; 4, early hyperfluorescence and late severely dye leakage which transit and beyond the borders of the laser burn lesion.

To determine the overall effect of a single injection of KH902 on the inhibition of laser-induced CNV, the areas of CNV for all time points were combined and averaged. This analysis demonstrated that 300 μg and 500 μg of KH902 inhibited growth of the neovascular area compared to findings in the control and animals treated with 100 μg KH902 (p<0.001 for 300 μg and 500 μg when compared with control and 100 μg). To assess the efficacy of KH902 over time, we measured growth of the CNV area beyond the laser spot on day 20 after laser injury and on days 14 and 28 after intravitreal injection. The results showed that on days 14 and 28, the area of neovascularization was significantly lower in the 300 μg and 500 μg KH902-treated eyes compared to the control and 100 μg KH902-treated eyes (p<0.001; Figure 3). Thus, with one injection of KH902, neovascular growth remained inhibited throughout the 28-day follow-up period.

**Figure 4:**
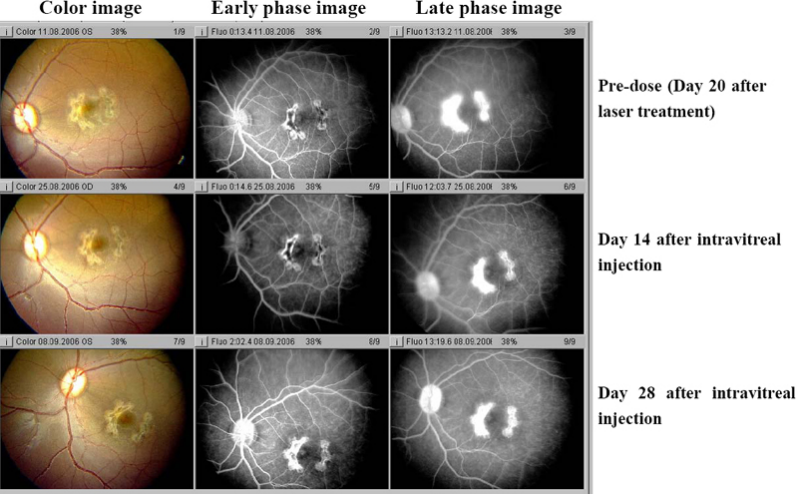
Color photography and angiography of an eye that received injection of vehicle taken on day 20 after laser and on days 14 and 28 after intravitreal injection. Color photo and fluorescein angiography of experimental model of choroidal neovascularization treated with injection of vehicle is shown. Note the gray-white change of lesions, local retina edema on color image and the large confluent bridging lesions and extensive late leakage on angiography image in left eye. There were no changes among three time points: day 20 after laser, day 14 and 28 after intravitreal injection.

**Figure 5:**
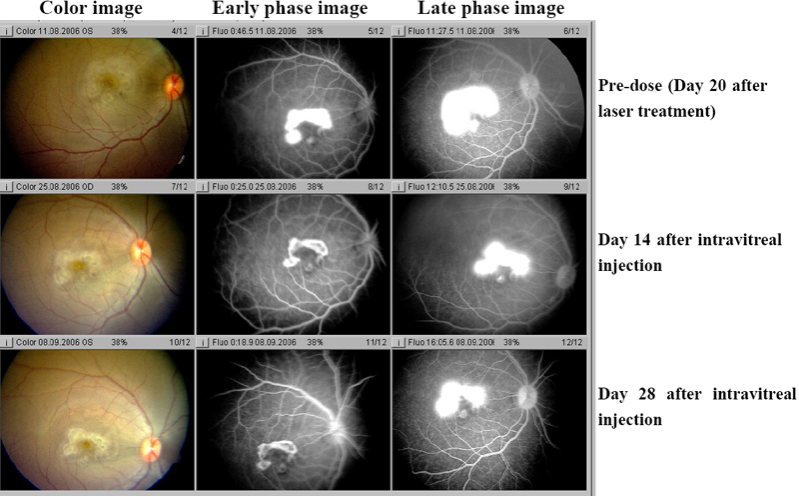
Color photography and angiography of an eye that received injection of 100 μg KH902 taken on day 20 after laser, days 14 and 28 after intravitreal injection. Color photo and fluorescein angiography of experimental model of choroidal neovascularization treated with injection of 100 μg KH902 is shown. Note the gray-white appearance of lesions and local retina edema on color image. The large confluent bridging lesions and extensive late leakage were shown on angiography image of right eye on day 20 after laser. On days 14 and 28 after intravitreal injection, laser lesion and late leakage decreases a little but still kept extensive late leakage.

**Figure 6:**
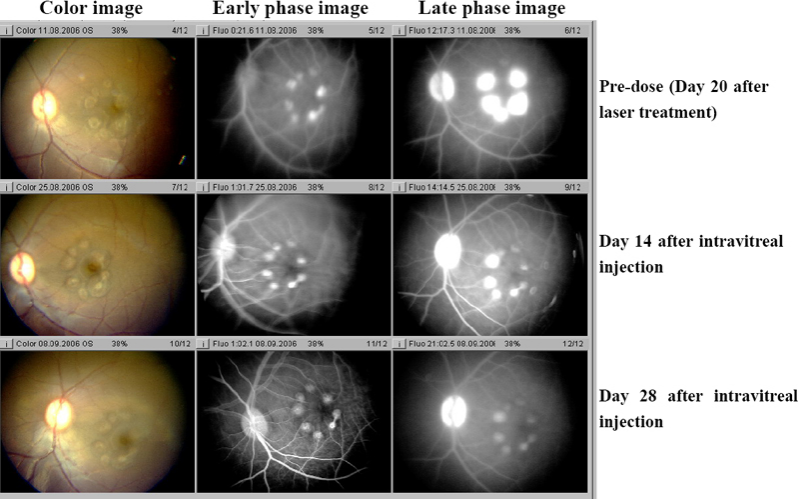
Color photography and angiography of an eye that received injection of 300 μg KH902 taken on day 20 after laser and on days 14 and 28 after intravitreal injection. Color photo and fluorescein angiography of experimental model of choroidal neovascularization treated with injection of 300 μg KH902 is shown. Note the gray-white appearance of lesions and local retina edema in the color image. The large confluent bridging lesions and extensive late leakage were demonstrated on angiography image in left eye on days 20 after laser. On day 14 after intravitreal injection, local edema and late leakage of lesions decreased. Furthermore, on day 28 after intravitreal injection, there was no evidence of retina edema and late leakage.

**Figure 7:**
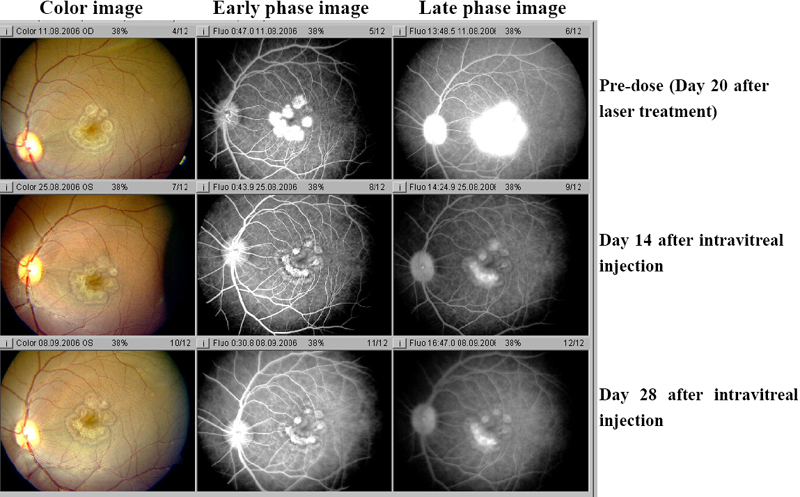
Color photography and angiography of an eye that received injection of 500 μg KH902 taken on day 20 after laser and days 14 and 28 after intravitreal injection. Color photo and fluorescein angiography of the experimental model of choroidal neovascularization treated with injection of 500 μg KH902 is shown. Note the gray-white appearance of lesions and local retina edema on color image. The large confluent bridging lesions and extensive late leakage were demonstrated on angiography image in left eye on days 20 after laser.

In addition to size, a single injection of KH902 was capable of decreasing the leakage. Both early and late fluorescein angiograms taken on days 14 and 28 demonstrate that leakage was inhibited in KH902-treated eyes. The eye that received an injection of the vehicle demonstrated a large confluent bridging lesion and extensive late leakage (Figure 4 and Figure 5). In contrast, an eye treated with 300 μg of KH902 or 500 μg of KH902 demonstrated not only an imperceptible increase in size of the laser lesion but also no late leakage of any of the laser spots (Figure 6 and Figure 7).

Through analysis of angiograms at each dose level, the proportion of spots that had clinically significant leakage as defined by a grade of 4 could be calculated for each time point (Table 1). On day 20 after laser burn, the control eyes had as much as 68.75%±44.19% of spots with active leakage as compared to 53.13%±25.77%, 53.13%±25.77%, and 71.88%±27.72% of spots in eyes injected with 100 μg, 300 μg, and 500 μg, respectively, of KH902. On day 14 after intravitreal injection, the percentage of spots with leakage decreased to 12.5%±25% and 6.25%±12.5% in 300 μg and 500 μg KH902-treated eyes, respectively, as compared to 62.5%±35.36% and 12.5%±25% in control and 100 μg KH902-treated eyes. The difference between eyes treated with 300 μg or 500 μg KH902 and eyes treated with 100 μg KH902 or buffer are still significant (p<0.05). On day 28 after intravitreal injection, the proportion of grade 4 spots in 300 μg and 500 μg KH902-treated eyes was 3.13%±6.25%. It was markedly decreased after intravitreal injection compared with that on day 20 after laser burn (p<0.001). In control and 100 μg KH902-treated eyes, the proportion of grade 4 spots were the same as it was on day 14.

**Table 1:** Comparison of the proportion of grade 4 spots in fluorescence fundus angiography between day 21 after laser burn and days 14 and 28 after intravitreal injection of KH902.

**Groups**	**Proportion of grade 4 spots on day 20 after laser**	**Proportion of grade 4 spots on day 14 after intravitreal injection**	**Proportion of grade 4 spots on day 28 after intravitreal injection**
Control	68.75±44.19	62.5±35.36	62.5±35.36
KH902 (0.1 mg)	53.13±25.77	12.5±25	3.13±6.25
KH902 (0.3 mg)	53.13±25.77	12.5±25*	3.13±6.25*
KH902 (0.5 mg)	71.88±27.72	6.25±12.5*	3.13±6.25*

The asterisk indicates that it is compared with the predose and the difference is significant using one way ANOVA analysis. When the difference among three groups is significant, post hoc dunnett was used to analyze the difference between the adminstration group and the predose group.

There was no apparent complication related to intravitreal injection of KH902. Throughout the experiment, no inflammation, cataract formation, retinal detachment, or vitreous hemorrhage was noted in any of the animals. The intraocular pressure results showed no significant change between the baseline and the after injection time point in any of the treated or control animals.

To confirm that angiographically measured and graded lesions represented CNV, angiograms were correlated to OCT and histopathologic findings. OCT image and histopathology confirmed that angiographically measured CNV that stained or leaked represented subretinal neovascularization and that the extent of angiographically measured lesions corresponded to the histological edge of the lesions.

**Figure 8:**
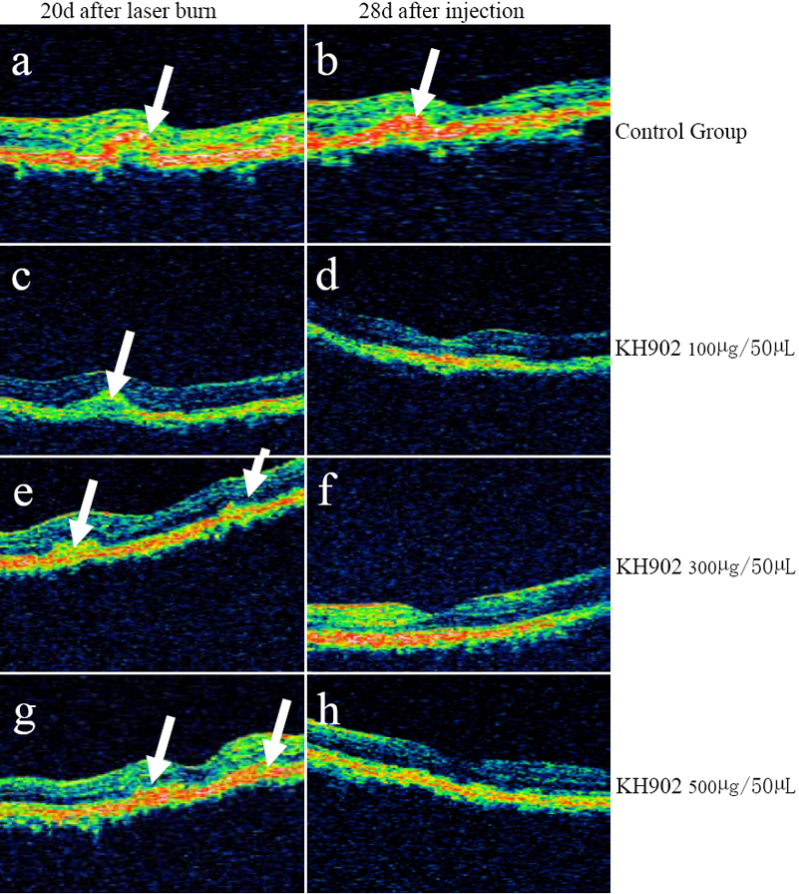
Optical coherence tomography scan image on day 20 after laser burn and day 28 after intravitreal injection in four groups. The high reflect light echogenic mass (white arrowheads) were detected in the eyes on day 20 after laser burn and in the eye of control on day 28 after intravitreal injection. The light echogenic mass disappeared after injection compared with that before injection. The light echogenic band of retinal pigment epithelial became regular and continuous. Edema of the retina faded away and the shape of the macula fovea centralis recovered.

On day 20 after laser burn, the high reflect light echogenic masses similar to human pathologic CNV were detected in all animals eyes. Neuroretina edema and abnormal macular fovea due to the CNV were demonstrated by OCT scan image. In the control and 100 μg KH902-treated eyes, there were no obvious changes on days 14 and 28 after intravitreal injection (Figure 8A-D). However, a marked change was detected in 300 μg and 500 μg KH902-treated eyes on days 14 and 28 after intravitreal injection. The light echogenic mass disappeared on the OCT scan image. The light echogenic band of retinal pigment epithelial became regular and continuous. Edema of the retina faded away and the shape of macula fovea centralis recovered (Figure 8E-H).

**Figure 9:**
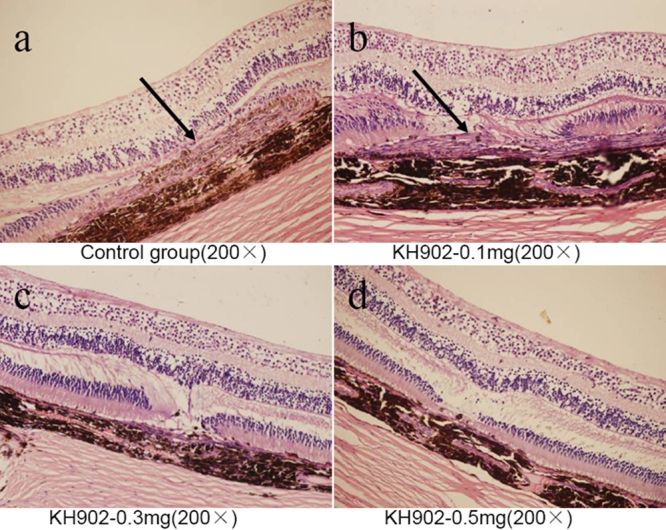
Ocular histology (hematoxylin-eosin stain) of all four groups at the endpoint of the experiment. The subretinal hyperblastosis (black arrowheads) in the control group and 100 μg KH902-treated group is shown. The outer nuclear layer was absent and retinal edema was obviously exhibited (**A**,**B**). In the 300 μg and 500 μg KH902-treated eyes, there were distinct retinal structures and no edema. The absent outer nuclear layer and a few fibrocytes were observed (**C**,**D**).

**Figure 10:**
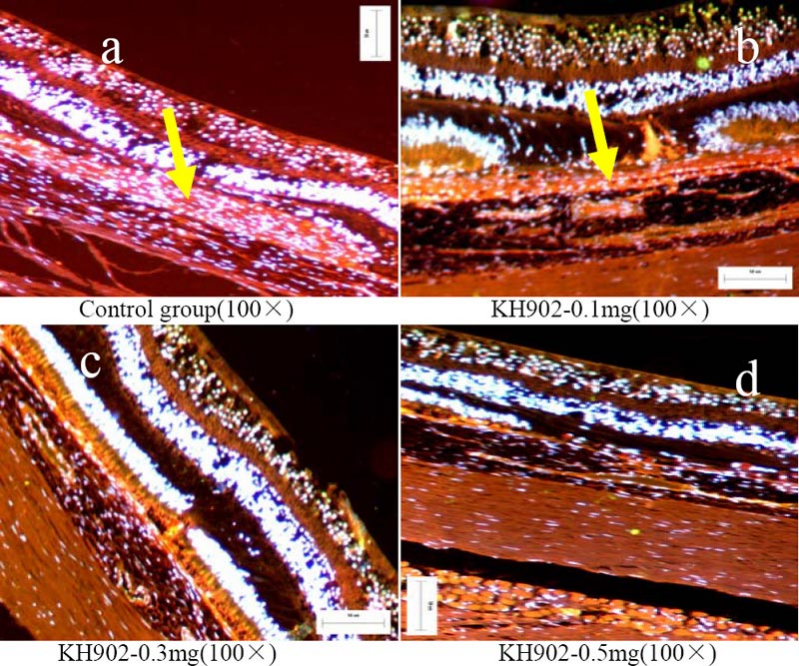
Merged images of immunohistochemistry. Immunohistochemistry analysis of proliferation-associated endothelial cells (stained by CD31/CD105 and DAPI) in all four groups at the endpoint of the experiment is shown. The proliferation-associated endothelial cells of choroidal neovascularization were stained with CD31 and CD105 (yellow arrowheads) like choroidal vessel endothelial cells in the control eye and 100 μg KH902-treated eye (**A**,**B**) whereas no positive stained proliferation-associated endothelial cells were found in 300 μg and 500 μg KH902-treated eye (**C**,**D**).

Histology analysis results indicated rupture of Bruch's membrane and subretinal hyperblastosis in the control group eye section and in the 100 μg KH902-treated eye section(Figure 9A,B). The proliferation-associated endothelial cells stained with CD31 and CD105 (Figure 10A,B), retinal pigment epithelial cells, macrophagus, and fibrocytes were seen under microscopy (Figure 9A,B). The absence of an outer nuclear layer and retinal edema were obvious. In the 300 μg and 500 μg KH902-treated eye sections, there were distinct retinal structure and no edema. The absent outer nuclear layer and a few fibrocytes were observed (Figure 9C,D), but there were no proliferation-associated endothelial cells stained with CD31 and CD105 (Figure 10C,D).

### KH902 improved the local multifocal electroretinogram of the macula involved by CNV in the monkey

**Figure 11:**
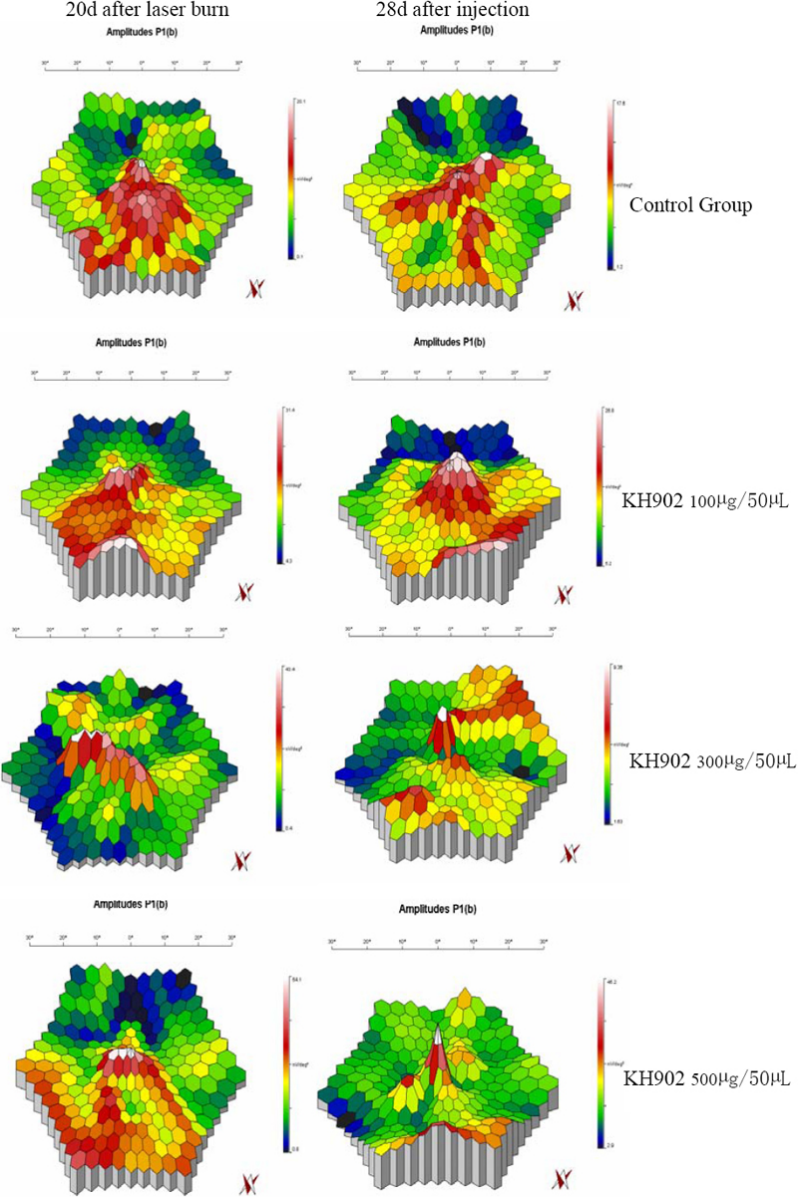
Local multifocal electroretinogram measurements on day 20 after laser and on day 28 after intravitreal injection. On day 20 after laser burn, the peak of the macular and the amplitudes of the retina that corresponded to those involved in CNV was markedly cut down. On day 28 after injection, the amplitudes increased and the function of locally involved retina improved compared to the results on day 20 after laser burn in the KH902-treated eyes, especially in the 300 μg and 500 μg KH902-treated one. But, there was no marked change in the control eye.

Local mf-ERG measurement allows for mapping of retinal function, and the location of dysfunction in the retina can be indicative of its future potential impact on vision. On day 20 after laser burn, the three dimensional map of mf-ERG demonstrated that the peak of the macular and the amplitudes of the retina corresponding to those involved in CNV cut down markedly. Local dysfunction in the macular retina could be presented clearly. In the control eyes, the abnormality presented no change on days 14 and 28 after intravitreal injection. However, in treated eyes on day 20 after laser, the amplitudes increased and the function of locally involved retina improved. Especially on day 28, the peak of the macular recovered and the map of the macular retina improved in those that received 300 μg and 500 μg KH902 (Figure 11).

## Discussion

The process of angiogenesis is multi-factorial and highly complex, but vascular endothelial growth factor (VEGF) is considered critical both in physiologic and in pathological angiogenesis [13] such as in AMD [14,15]. Binding of VEGF-A to its receptors leads to endothelial cell proliferation and neovascularization as well as vascular leakage, all of which are thought to contribute to the progression of the neovascular (wet) form of age-related macular degeneration, one of the leading causes of legal blindness. There are also a considerable number of publications showing that by antagonizing VEGF, angiogenesis and vascular permeability can be prevented [11,12,16-18].

There are currently two inhibitors of VEGF, Pegaptanib and Ranibizumab, which are approved by the FDA for the treatment of exudative AMD and under commercial development. Both of them could inhibit the ocular neovascularization efficiently, but frequent administration was required to maintain the therapeutic level of VEGF inhibition. Furthermore, frequent administration, which requires patients to be injected every four to six weeks, has been performed in clinical trials [11,12]. These limitations provide an opportunity to develop better VEGF inhibitors that can potentially be administered less frequently and act more potently.

VEGF-Trap, a unique VEGF blocker, is a fusion protein of key domains from VEGF receptor 1 and 2 with human IgG Fc. It has a high affinity with VEGF and blocks all VEGF-A isoforms and placental growth factors. It can also penetrate all layers of the retina. The interim results of the Phase II clinical trial show that the mean change from the baseline in visual acuity demonstrated statistically significant improvement (all groups combined increase 5.9 letters, p<0.0001), and monthly and quarterly dosing did not result in substantially different results at eight weeks [19].

In the current study, we designed a molecule KH902, similar to VEGF-Trap. It combines ligand binding elements taken from the extracellular domain 2 of VEGF receptors 1(Flt-1) and extracellular domain 3 of VEGF receptors 2 (KDR) fused to the Fc portion of human IgG1. The molecule still contains a extracellular domain 4 of VEGF receptors 2(KDRd4), which can improve the three-dimensional structure and increase the dimerization efficiently [20,21] so that KH902 presents high affinity with VEGF. By human VEGF ELISA assay, the IC_50_ of KH902 was 7–15 pM and the mean IC_50_ was 10 pM. Compared with avastin, an off-label anti-VEGF drug which was administrated to treat wet-AMD in fashion, the affinity of KH902 was 50 fold times more than that of avastin and equally more efficient in inhibiting the proliferation of human umbilical vein endothelial cells induced by VEGF. Meanwhile, KDRd4 degrades the isoelectric point (PI) of KH902, which maintain the soluble state of the active proteins and may thus prolongs the clearance time of KH902 in the vitreous. We will confirm the hypothesis in later pharmacokinetic experiment.

Furthermore, we sought to demonstrate the efficacy of KH902 in a clinically relevant model of CNV. What we have shown in this study is that a single intravitreal injection of KH902 targeting VEGF can prevent growth of CNV and attenuate vascular leakage of CNV for at least four weeks. A single injection of our high dose KH902 suppressed leakage throughout the follow-up period, indicating that the duration of action on vascular permeability with one injection of KH902 is at least four weeks, especially at our higher dose. At the dose levels used in this study, no evidence of inflammation or toxicity was noted. Formal animal toxicity studies are necessary to determine the highest acceptable dose level that can be delivered to the eye. This study illustrates the potency of KH902 as a therapeutic class of molecule. As a therapeutic agent, KH902 is potent, highly soluble, shows no toxicity, and appears to have an effective suppression action on CNV.

The stimulus for neovascularization in laser-induced lesion models obviously differs from that in AMD because laser models invoke a traumatic repair process that may mimic traumatic CNV development better than AMD-related CNV. However, there are also substantial similarities between AMD-related CNV and laser-induced CNV. Investigators using a primate laser model have postulated that macrophages, involved in the initial response to Bruch's membrane injury after laser exposure, secrete angiogenic growth factors. These growth factors are probably mechanistically relevant to human CNV formation as surgically excised and postmortem CNV and RPE cells in human tissues have shown immunoreactivity for these same growth factors.

In our study, we used the mf-ERG examination to demonstrate locally involved macular function variation during the experiment. The results of mf-ERG demonstrated the improvement was greater in 300 μg and 500 μg KH902-treated eyes than in the 100 μg KH902-treated eyes and eyes of the control group. These results imply that the central vision of monkey eyes impaired by laser-induced CNV obtained improvement after intravitreal injection of KH902.

In summary, KH902, the molecule with a high affinity binding for VEGF, effectively inhibits the proliferation of human umbilical endothelial cells induced by VEGF in vitro. A single intravitreal administration of KH902 successfully prevented lesion growth and leakage of choroidal neovascular in a nonhuman primate model. In addition, this preclinical proof of principle provides a guideline for dosing in humans and provides strong support for advancing this molecule into clinical trials for the treatment of wet age-related macular degeneration.
